# Numeric Error in Table 1

**DOI:** 10.1001/jamanetworkopen.2021.23147

**Published:** 2021-07-23

In the Original Investigation titled “Yield and Utility of Germline Testing Following Tumor Sequencing in Patients With Cancer,”^1^ published October 7, 2020, there was a numeric error in Table 1. The percentage of female patients given as 56.3 should have been 53.6. This article has been corrected.^1^
